# Supplementary material for: Can head louse repellents really work? Field studies of piperonal 2% spray
Source: PeerJ. 2014 Apr 17;2:e351. doi: 10.7717/peerj.351 (PMC3994642; doi:10.7717/peerj.351)
Supplement: Supplemental Information 2 [file peerj-02-351-s002.pdf]

CHARWELL
PHARMACEUTICALS

STUDY PROTOCOL

Charwell Pharmaceuticals Ltd
Charwell House Wilsom Road Alton Hampshire GU34 2TJ Telephone (0420) 84801 Fax (0420) 89376
[illegible]

RAPPELL LOUSE REPELLENT FIELD STUDY

Sponsor: Charwell Pharmaceuticals Ltd
Charwell House
Wilsom Road
Alton
Hants GU34 2TJ

Contact: Mr W J Oliver

Principal Investigator: Ian Burgess, BSc, MSc, MPhil, FRSH
Medical Entomology Centre at the University of Cambridge

Supervising Doctor: Dr J S Adler, MB, BS, Dip ABIM
682 Finchley Road
London NW11 7NP

Supervising Professionals and Study Co-ordinators:

Christine Brown, RGN, SNCert
Medical Entomology Centre at the University of Cambridge

Judith Kaufman, BPharm, MRPharmS
55 Templars Avenue
London NW11 ONU

Proposed date of commencement: April 1994

Proposed date of completion: August 1994

CONTENTS

PERSONNEL

STUDY FACILITIES

1. INTRODUCTION
2. OBJECTIVE AND STUDY DESIGN
3. DRUG SUPPLY
4. DRUG DISPENSING
5. ETHICAL ASPECTS
   - 5.1 Ethical Review
   - 5.2 Information for Volunteers
   - 5.3 Informed Consent
   - 5.4 Notification for General Practitioner
   - 5.5 Confidentiality
   - 5.6 Indemnity
6. SELECTION OF VOLUNTEERS
   - 6.1 Inclusion Criteria
   - 6.2 Exclusion Criteria
   - 6.3 Pre-study medical requirements
7. STUDY SCHEDULE
   - 7.1 Study Period
   - 7.2 Study Groups
8. RESTRICTIONS ON VOLUNTEERS
   - 8.1 Concomitant Treatment
   - 8.2 Reapplication of Product

CONTENTS (continued)

9. REPLACEMENT OF VOLUNTEERS

10. ADVERSE EVENTS

11. STATISTICAL METHODS

12. RECONCILIATION OF STUDY SUPPLIES

13. DATA COLLECTION AND REPORTING

14. QUALITY ASSURANCE PROCEDURES

15. STUDY MONITORING

16. ARCHIVING DATA

17. PROTOCOL AMENDMENTS

APPENDICES

1. Information Document

2. Volunteer Informed Consent Form

3. Group 1 Diary Card

4. Groups 2 and 3 Diary Card

5. Primary Volunteer Report Card

6. Randomisation Procedure

PERSONNEL

Sponsor:	Charwell Pharmaceuticals Ltd<br>Charwell House<br>Wilsom Road<br>Alton<br>Hants GU34 2TJ
Approved by:	W J Oliver, BA, MBA
	Signature: ....
	Title: Technical Director
	Date: ....
Principal Investigator:	I Burgess, BSc, MSc, MPhil, FRSH<br>Medical Entomology Centre
	Signature: ....
	Date: ....
Supervising Professionals:	
Doctor:	Dr J S Adler, MB, BS, Dip ABIM
	Signature: ....
	Date: ....
Study Co-ordinators: i)	C Brown, RGN, SNCert<br>Medical Entomology Centre
	Signature: ....
	Date: ....
ii)	J Kaufman, BPharm, MRPharmS
	Signature: ....
	Date: ....
Quality Assurance:	E P Hitchins, CChem, MRSC<br>Charwell Pharmaceuticals Ltd
	Signature: ....
	Date: ....
Statistical Advisor:	Peter C Jones, MSc<br>BIOS (Consultancy & Contract Research) Ltd
	Signature: ....
	Date: ....

STUDY FACILITIES

FIELD PHASE

Centred on
Beis Yaakov Primary School
Edgeware Road
Colindale
London NW9

Investigation Centre
Medical Entomology Centre of the
University of Cambridge

Statistical Evaluation
BIOS (Consultancy & Contract Research) Ltd

1. INTRODUCTION

Rappell is a novel head louse repellent containing Piperonal at 2% w/w as the active ingredient. Piperonal (3,4-Methylenedioxybenzaldehyde, C.A.S. registry number 120-57-0) is used extensively in foods, perfumes and household products. Piperonal was granted GRAS (Generally Recognised As Safe) status and approved for food use by the U.S. Food and Drug Administration in 1965. The compound was listed by the Council of Europe in 1970 to be approved for food use with an ADI (Acceptable Daily Intake) of 2.5 mg per kg body weight. The Joint FAO/WHO Expert Committee on Food Additives evaluated the compound in 1977 (Food Legislative Survey No. 2, BFMIRA (1977)) and also agreed/accepted an ADI for Piperonal of 2.5 mg per kg body weight.

Head lice, *Pediculus humanus capitis* are of cosmopolitan distribution with few areas of the world where they are unknown. They are parasites of the human scalp from which they suck blood as their food, thereby inducing an allergic reaction in the host as a response to saliva injected into the wound. Such immune responses to saliva may take some time to develop.

These insects reproduce by laying eggs that the female louse cements firmly onto hairs in close proximity to the scalp where it is warm enough for them to hatch. The eggs take approximately six or seven days to complete their development prior to emergence of the nymphal lice. Louse nymphs are like miniature adults and behave in essentially the same way. They undergo three moulting stages (instars) during their growth which takes about two weeks under normal conditions. Each of the development stages is distinguishable by its size and shape. Adults mature in approximately three days following the final moult after which they can mate successfully. Females will continue to lay eggs for the remainder of their lives up to about 28 days. The principal infective stages are considered to be a nuisance, rather than life threatening. It is only in the developed world that strenuous efforts have been made towards their eradication by diversion of resources, health education and the development of a variety of chemical treatment formulations. The newest development, and one which consumers have been demanding for some time, is the formulation of a preventative to be used by people who wish to guard against catching head lice or who, having successfully treated an infection, wish to avoid the risk of a repetition.

2. OBJECTIVE AND STUDY DESIGN

This field study is designed primarily to establish whether the louse repellent Rappell statistically reduces the risk in healthy volunteers exposed to a significant potential for head louse infection. This field study will also determine whether the *in-vitro* repellency results are borne out under field conditions.

The study will be conducted in compliance with the OECD principles of Good Laboratory Practice (GLP) and Good Clinical Practice (GCP) for trials on medicinal products in the European Community.

The study will be conducted as a parallel group study consisting of three groups, each containing an equal number of children. Families expressing an interest will be invited to a preliminary meeting, with further workshops and/or seminars for those willing and able to participate in the study. Full written explanatory details will be given to each family.

Study Population

The area chosen for the study is centred on a Jewish Orthodox community in North London. They approached the Medical Entomology Centre seeking help in 1992/93 as the head louse problem in the community, centred on the school-age children particularly, appeared to be considerably higher than other areas of the United Kingdom. Careful monitoring over an eight month period has shown this to be true. One school in the area, Beis Yaakov Primary School, has a prevalence rate of 25% against a single figure average elsewhere in the country. It is the families with children at the Beis Yaakov Primary School, Colindale, and associated schools who will be the main source of volunteers. It is thought that these communities have a particularly high prevalence due to frequent trips of family members to Israel and other countries where head lice are endemic and the fact that they appear to have been bypassed by mainstream health education and services.

3. DRUG SUPPLY

The products will be supplied as follows in sufficient quantity for 100 volunteers per group. The products will be accompanied by Certificates of Analysis. Sufficient product for one month's usage will be provided on the first supervisor's check for each month. The remaining stock for each volunteer will be retained by the study coordinators or the sponsoring company for supply at the appropriate stage.

Active Product

Rappell Lotion manufactured to formula number 3/93/3 containing Piperonal at 2% w/w in an aqueous alcoholic base.

Supplier: Charwell Pharmaceuticals Ltd

Batch Number: [To be included as a protocol amendment]

Control Product

Placebo Rappell Lotion manufactured to formula number D9213 containing Vanillin at 1% w/w as an odour masking fragrance.

Supplier: Charwell Pharmaceuticals Ltd

Batch Number: [To be included as a protocol amendment]

4. DRUG DISPENSING

The test product or control product will be provided in packaged units containing 90 ml of product which should be sufficient for one month's use by one volunteer. The packaging presents the product in metered doses of 130 ul. Each packaged unit will be labelled with the volunteer's name or number, the study number, a coded sample identification number, method of application and all relevant safety information.

5. ETHICAL ASPECTS

5.1 Ethical Review

Details of the proposed study (final protocol, information sheet for volunteers and consent form) will be submitted to an independent ethics committee for their consideration, comments and guidance. Approval in writing must be received before the study commences. The study will be conducted in accordance with the principles of the Declaration of Helsinki (1964) and the Tokyo (1975), Venice (1983) and Hong Kong (1989) Revisions.

5.2 Information for the Parents of Volunteers

The purpose of the study, together with the methods, possible hazards and discomfort will be explained to the parent of each volunteer, both verbally and in writing. An information document will be issued to the parents of each prospective volunteer (Appendix 1). The parents of each volunteer will be informed that they are free to withdraw their child from the study at any time, but must comply with the directions of the supervising professional.

5.3 Informed Consent

The parent of each volunteer will read and sign an Informed Consent form (Appendix 2) which will be countersigned by a supervising professional and a witness to confirm that the form has been read and explained.

5.4 Notification of General Practitioner

Prior to commencement of the study, the General Practitioner of each subject will be notified of that volunteer's intended involvement in the study. The General Practitioner will be asked if there is any contraindication to the volunteer's participation. A written response to the effect that there is no contraindication must be received before the volunteer is admitted to the study.

5.5 Confidentiality

The name and personal details of each volunteer will be kept on file by the investigator. These details may be held on a computer database and the volunteers will be informed, as required by the Data Protection Act 1987, that these details are on file. Precautions will be taken by the investigator to ensure that unauthorised access to these records is not possible.

Personal details including name and address will be kept on original confidential records retained by the investigator. Case record forms will be identified only by a number, the initials of the volunteer and a summary of clinical details.

Authorised representatives of the sponsor who are monitoring the study may be given sight of original clinical records including consent forms which might identify the volunteer, but only for the purpose of verifying the authenticity of the records; no copies of such data will be supplied to the sponsor or a third party.

5.6 Indemnity

The sponsor will supply written agreement, based on ABPI guidelines, to indemnify the investigator against any claim for injury or illness attributable to product administration or study procedures provided the protocol is adhered to.

6. SELECTION OF VOLUNTEERS

Volunteers who fulfil the following criteria, will be considered for use in the study.

6.1 Inclusion Criteria

1. Aged between 4 and 14 years.
2. Normal physical health as judged by medical history.
3. A willingness to participate in the study.

6.2 Exclusion Criteria

1. Any history of significant allergic reactions especially asthma and eczema.
2. Any history of dermatological disorders such as contact dermatitis or psoriasis.
3. Any ongoing treatment liable to sensitise the subject such as steroid administration.
4. Any contraindication to participation in the study noted by the subject's physician.
5. Any subject found to be infected with head lice who is unwilling to undergo treatment to remove the infection.

6.3 Pre-study Medical Requirements

Assessment of medical history and general state of health by a physician.

7. STUDY SCHEDULE

7.1 Study Period

It is intended that the study commences during April 1994 and should continue for a minimum of six weeks and a maximum of 13 weeks. However there is no objection to the study being extended after this period if circumstances dictate.

Because of the large number of volunteers involved in this study and the practical aspects of carrying out the inspections, the study will be initiated in a rolling fashion.

Prior to the commencement of the study, all participants will be examined by a suitably qualified investigator to ascertain the absence of any head louse infection. Any participant found to be infected will be treated to remove the infection.

7.2 Study Groups

Group 1: Every participating child will be checked for head lice using a detection comb. The parents will be asked to carry out treatment with an aqueous based carbaryl liquid treatment (e.g. Derbac-C Liquid) if any evidence of head louse infection is found. This group will form the base-line control as they will receive no further chemical interference unless they become infected with head lice. Should infection occur, they will be treated as above.

Volunteers to Group 1 will be allocated according to the block randomisation schedule as described in Appendix 6.

Any volunteer wishing to continue after an infection has occurred and successful treatment has been given, can be transferred to either Group 2 or Group 3 but cannot continue in Group 1.

A Group 1 diary card (see Appendix 3) will be provided for each volunteer. A parent of each volunteer will be asked to complete the diary card following each inspection. The diary card will be collected at the end of each month or when the supervisors' visits are conducted.

Groups 2 and 3: Every participating child will be checked for head lice using a detection comb. The parents will be asked to carry out treatment with an aqueous based carbaryl liquid treatment (e.g. Derbac-C Liquid) if any evidence of head louse infection is found. The parents of the volunteer children will be given either the active product or the placebo product to spray onto the volunteer's hair daily.

The treatments will be issued according to a block randomisation schedule as described in Appendix 6. The statistical requirements of the study demand that families where members are involved in Groups 2 and 3 will often be provided with both active and placebo treatments. These treatments will be labelled clearly with the child's name or code number and must only be used on the named child.

These families will be asked to use the spray daily, usually at the same time each day. There may be instances when more than one daily spray is required, for instance when a child has been swimming the spray will need to be reapplied. A diary card will be issued for each family member in the study. For each month of the study one card will be issued, on which families will mark "X" each time the spray is used and "O" if they do not. It will be requested that any lice found during detection combing are stuck onto the card with clear adhesive tape. No other "preventative", "treatment lotions or shampoos" should be used for the duration of the study, but if they are their use should be recorded on the Group 2/3 diary card (Appendix 4).

Any volunteer wishing to continue after an infection has occurred and successful treatment has been given, can be transferred to Group 1 but cannot continue in either Group 2 or Group 3.

8. RESTRICTIONS ON VOLUNTEERS

8.1 Concomitant Treatment

Volunteers are required to avoid the use of any treatments to clear lice infections or prevent lice infection during the entire study period other than that issued by the Study Co-ordinators. Any treatment of this nature should be noted on the diary cards and reported to the investigator as soon as possible.

8.2 Reapplication of Product

Following any process likely to remove the product from the hair, e.g. swimming, showering after sports activities, the product should be reapplied to the dried hair.

9. REPLACEMENT OF VOLUNTEERS

Any volunteer withdrawing from the study will be replaced such that statistically required number of volunteers complete the study.

10. ADVERSE EVENTS

As this study is being performed on a marketed product no significant adverse events are expected. Any adverse events reported spontaneously or following questioning will be reported to the Supervising Doctor for assessment. All adverse events considered to be related to the materials provided during the study and judged to be significant by the Supervising Doctor will be recorded and reported to the Sponsors and the Chairman of the Ethics Committee.

11. STATISTICAL METHODS

The data produced by this field study will be subjected to that statistical analysis deemed appropriate by the Consultant Statistician. The results of the statistical analysis will be reported to the Principal Investigator and the Sponsors.

12. RECONCILIATION OF STUDY SUPPLIES

An inventory of all study supplies received and dispensed will be prepared by the Study Co-ordinators and supplies not used will be returned to the Sponsors.

All treatment packs will be returned at the end of each month and replaced with a fresh pack. The treatment packs will be weighed prior to dispensing and upon return to check compliance with recommended usage rates.

13. DATA COLLECTION AND RECORDING

All data will be collected on diary cards and on the primary volunteer report card, copies of which are shown in the Appendices 3, 4 and 5 to this Protocol. These records will be kept by the Medical Entomology Centre at the University of Cambridge. The raw data contained on the forms will be sent to the Consultant Statistician for analysis. The Principal Investigator will prepare and submit a detailed report of the study in accordance with Good Clinical Practice.

14. QUALITY ASSURANCE PROCEDURES

All aspects of the study will be subjected to a Quality Assurance Assessment by the designated professional, including a field audit to assess compliance in data recording. The final study report will be fully audited for compliance with Good Clinical Practice.

15. STUDY MONITORING

Personnel from the Sponsoring Company will be granted access to the Medical Entomology Centre facility to inspect data records and to observe the field study procedures.

16. ARCHIVING

A copy of the final report, volunteer records and diary cards will be stored with the Medical Entomology Centre or the Sponsors for a period of 15 years. Following this a decision will be made, by the Sponsors, whether to retain or destroy the data.

17. PROTOCOL AMENDMENTS

If an amendment is required after submission to the Ethics Committee, this will be enacted through a formal amendment procedure. If amendments are such as to raise ethical issues, the amendments will be submitted to the Chairman of the Ethics Committee who has the right to submit the amendments to all members of the original Committee prior to giving approval.

APPENDIX 1

INFORMATION DOCUMENT

INFORMATION DOCUMENT

1. The company sponsoring the study is CHARWELL PHARMACEUTICALS LTD.

2a. The supervising doctor is Dr J S Adler, MB, BS, DipABIM.

2b. The supervising professionals are:

Ian Burgess, BSc, MSc, MPhil, FRSH
Christine Brown, RGN, SNCert
Judith Kaufman, BPharm, MRPharmS

3. The principal features of the study are:

   i) Regular inspection of children's heads for the presence of head lice by the child's parent and/or a suitably qualified professional investigator.

   ii) Daily application to the hair of the marketed product Rappell or a scented placebo by the child's parent and/or a suitably qualified professional investigator, or no application depending on the study group entered.

   iii) Careful recording of the application and inspection results on a diary card by the child's parent and/or a suitably qualified professional investigator.

4. If assistance or advice is required in an emergency please use the following contact telephone numbers:

(0420) 84801	Monday to Friday 09:00 to 17:00
(0420) 82813	At all other times (24 hour answer phone giving alternative telephone number)

5. Some insurers treat participation in medical studies as a material fact which should be mentioned when making any proposal for health-related insurance and that accordingly participation in the study should be disclosed if the volunteer is in the process of seeking or renewing any such insurance and the volunteer should check that participation does not affect any existing policies held by the volunteer.

6. The study has been subject to review by an independent ethics committee.

7. The volunteer must inform the supervising professional or doctor of the following:

   i) Any illness arising during the course of this study.

   ii) Any abnormal occurrences thought to be due to the materials used in the study.

   iii) The use of any louse treatment or louse repellent other than that supplied by the supervising professionals.

   iv) Any intention to discontinue the study.

APPENDIX 2

VOLUNTEER INFORMED CONSENT FORM

VOLUNTEER AGREEMENT AND CONSENT FORM

1. I, the undersigned, voluntarily agree to my child/children, named in this document, participating in a louse repellent field study.

2. I have been given a full explanation by the supervising professional, of the nature, purpose and likely duration of the study and what my child/children will be expected to do and I have been advised about any discomfort and possible ill-effects on my child/children's health or well-being which they believe may result. The information document given to me is attached.

3. I have been given the opportunity to question the supervising professional on all aspects of the study and have understood the advice and information given as a result.

4. I agree to the supervising professional contacting my child/children's general practitioner to make known their participation in the study and I authorise my child/children's general practitioner to disclose details of their relevant medical or drug history, in confidence.

5. I agree to comply with any instruction given during the study and to co-operate faithfully with the supervising professionals and to tell them immediately if my child/children suffers any deterioration of any kind in his/her health or well-being or any unexpected or unusual symptoms however they may have arisen.

6. I agree that I will not seek to restrict the use to which the results of the study may be put and, in particular, I accept that they may be disclosed to regulatory authorities for medicines in the UK and elsewhere.

7. I understand that I am free to withdraw my child/children from the study at any time without needing to justify my decision.

8. The company sponsoring the study confirms that:

   i) As I am willing for my child/children to participate in the study voluntarily, I shall not receive any payment.

   ii) Subject to any overriding requirement of law necessitating the disclosure of documents relating to the study, the volunteer will not be referred to by name in any document concerning the study disclosed to any person not under the direct control of the supervising doctor or scientist;

   iii) In the event of my child/children suffering any significant deterioration in health or well-being caused directly by his/her participation in the study, compensation will be paid to me by the company.

   iv) The amount of such compensation shall be calculated by reference to the amount of damages commonly awarded for similar injuries by an English court if liability is admitted, provided that such compensation may be reduced to the extent that I, by reason of contributory fault, am partly responsible for the injury (or where I have received equivalent payment for such injury under any policy of insurance effected by the company for my child/children's benefit);

   v) Any dispute or disagreement as to the application of clause 8 (iii) shall be referred to an arbitrator to be agreed between myself and the company, or in the absence of agreement, to be appointed by the President of the Royal College of Physicians of London with power in the arbitrator to consult a barrister of 10 years' standing in respect of any issue of law including the amount of damages to be awarded as payment of compensation.

9. The agreement shall be construed in accordance with English law and, subject to clause 8 (iii), (iv) and (v) above, the English courts shall have sole jurisdiction over any dispute which may arise out of it.

Name(s) of children: ....

....

....

....

Name and address of General Practitioner: ....

....

....

....

Signed by (parent): ....

Dated: ....

Signed for and on behalf of
the Company by
its duly authorised
representative ....

Dated: ....

I confirm that I have explained the scientific and medical nature, purpose and possible hazards involved in this study to the above named parent.

Signed ....

I confirm that I have witnessed the above explanation.

Signed ....

APPENDIX 3

GROUP 1 DIARY CARD

REPELLANTS AGAINST HEADLICE
DIARY CARD

MONTH		SUBJECT NAME	
CARD NUMBER		TRIALIST NUMBER	

SUNDAY	MONDAY	TUESDAY	WEDNESDAY	THURSDAY	FRIDAY	SATURDAY

IMPORTANT

You are part of a control group. Please place a tick in the day box of any day in which you carried out an inspection with the headlice detector comb. If you find any trace of lice or eggs please remove and stick lice or eggs in day box and **INFORM YOUR INVESTIGATOR** before commencing any treatment.

APPENDIX 4

GROUPS 2 AND 3 DIARY CARD

REPELLANTS AGAINST HEADLICE
DIARY CARD

MONTH		SUBJECT NAME		TREATMENT CODE	
CARD NUMBER		TRIALIST NUMBER			

SUNDAY	MONDAY	TUESDAY	WEDNESDAY	THURSDAY	FRIDAY	SATURDAY

IMPORTANT
Please record each day that the spray is use with an **X** in the day box. If you need to spray more than once, i.e. after swimming, please record each additional spraying with an additional **X** . Please record an **O** in the day box if you did not spray on that day. Please place a tick in the day box of any day in which you carried out an inspection with the headlice detector comb. If you find any trace of lice or eggs please remove and stick lice or eggs in day box and **INFORM YOUR INVESTIGATOR.**

DIARY CARD GROUP 2 and 3

APPENDIX 5

PRIMARY VOLUNTEER REPORT CARD

REPELLANTS AGAINST HEADLICE PRIMARY RECORD CARD

TRIALIST NUMBER	TREATMENT CODE	GROUP	DATE OF SUBJECT COMMENCEMENT

PERSONAL DETAILS [CONFIDENTIAL]

SUBJECT FAMILY NAME { }

SUBJECT FIRST NAME { }

SUBJECT MIDDLE NAME { }

SEX OF SUBJECT Male [ ] Female [ ]

AGE OF SUBJECT Years [ ] Months [ ]

ADDRESS OF SUBJECT { }
{ }
{ }
{ }
{ }

HAIR LENGTH Above Ears [ ] Ears to Shoulder [ ] Below Shoulder [ ]

NUMBER OF ADULTS IN FAMILY Male [ ] Female [ ]

NUMBER OF SIBLINGS MALE [ ] Female [ ]

SIBLING TRIAL NUMBERS (if in trial) { }

RELEVANT MEDICAL HISTORY

NAME AND ADDRESS OF SUBJECTS G.P. { }
{ }
{ }
{ }
{ }

PARENTAL CONSENT FORM OBTAINED ON [ ] BY [ ]

IMARY CARD (FRONT)

REPELLANTS AGAINST HEADLICE PRIMARY RECORD CARD

DATE	BOTTLE NUMBER	INITIAL PACK WEIGHT (gm)	RETURNED PACK WEIGHT (gm)	USED (gm)	DIARY CARD RETURNED	MONITORS INITIALS

DATE	NOTES	MONITORS INITIALS

IMARY CARD (BACK)

APPENDIX 6

RANDOMISATION PROCEDURE

Sample Randomisation

Randomisation "groups" will be allocated to families. The first two digits refer to the family group. Within that group there will be eight treatments. The third digits will give the patient number (1 to 96) which should be allocated sequentially within each age group. The patients that are randomised to receive no treatment will be indicated by the letters NT and may be omitted if sufficient patients have been recruited to that group at the time. As a consequence numbers may be missing from some sequences within families. As these will only be "not treated" patients this will not be a problem.

Unused medication should not be given to other families. Families should not be allocated more than one per "group".

Actual sample randomisation table will be randomly generated by computer on confirmation of estimated number of groups, i.e. family units involved in the trial.

Example

Code	Explanations
01 1	Family group 01, subject number 1
01 2NT	Family group 01, subject number 2 allocated "not treated", can be omitted
01 3	Family group 01, subject number 3
01 4	Family group 01, subject number 4
01 5	Family group 01, subject number 5
01 6NT	Family group 01, subject number 6 allocated "not treated", can be omitted
01 7	Family group 01, subject number 7
01 8NT	Family group 01, subject number 8 allocated "not treated", can be omitted
01 9	Family group 01, subject number 9
02 1	Family group 02, subject number 1 etc
